# Circulating Muscle Markers for Sarcopenia in Peritoneal Dialysis

**DOI:** 10.1155/ijne/8817404

**Published:** 2025-10-15

**Authors:** Lixing Xu, Jack Kit-Chung Ng, Winston Wing-Shing Fung, Gordon Chun-Kau Chan, Kai-Ming Chow, Cheuk-Chun Szeto

**Affiliations:** ^1^Carol & Richard Yu Peritoneal Dialysis Research Centre, Department of Medicine & Therapeutics, Prince of Wales Hospital, Hong Kong, China; ^2^Li Ka Shing Institute of Health Sciences (LiHS), Faculty of Medicine, The Chinese University of Hong Kong, Shatin, Hong Kong, China

**Keywords:** frailty, malnutrition, renal failure

## Abstract

Sarcopenia, characterized by the progressive decline in muscle mass and function, is an important complication among patients undergoing peritoneal dialysis (PD). Despite its prevalence, there is a notable lack of comprehensive research focused on early diagnosis or treatment target for sarcopenia in PD patients. This review explores the pathophysiology of sarcopenia and examines the potential role of various biomarkers related to muscle mass and function. These markers include myokines (cytokines produced by skeletal muscle) such as myostatin, follistatin, activin A, growth differentiation factor 15 (GDF-15), and irisin, as well as adiponectin, leptin, insulin-like growth factor-1 (IGF-1), vitamin D, myoglobin, and cortisol. These markers hold promise for early diagnosis and as therapeutic targets for managing sarcopenia. By reviewing the current literature in this area, we aim to identify directions for further research on diagnostic strategies and therapeutic options for sarcopenic PD patients.

**Summary**

• Sarcopenia, involving a decline in muscle mass and function, is common in peritoneal dialysis patients.
• We reviewed the pathophysiology and highlighted biomarkers, including myokines, IGF-1, and vitamin D for early recognition of sarcopenia.

## 1. Introduction

End-stage renal disease (ESRD) is a serious and costly chronic disease. Compared to traditional hemodialysis, peritoneal dialysis (PD) offers several distinct advantages, including superior preservation of residual kidney function, enhanced lifestyle flexibility, cost-efficiency, and improved quality of life [1]. However, patients undergoing PD may encounter significant complications that can adversely impact their prognosis and quality of life. Of particular concern is sarcopenia, characterized by the loss of skeletal muscle mass, which substantially affects both the general population [2, 3] and patients with cancer [2, 3], but more commonly on dialysis patients [4].

The optimal technique for the assessment of muscle mass and recognition of sarcopenia remains controversial. Emerging evidence suggests that several biomarkers may serve as surrogate markers of muscle mass and function. These biomarkers have the potential to aid in the early diagnosis of sarcopenia and may represent promising therapeutic targets for the treatment of sarcopenia. Here, we review the current literature on muscle biomarkers in PD patients.

## 2. Sarcopenia

### 2.1. Definition of Sarcopenia

In 2010, the European Working Group on Sarcopenia in Older People (EWGSOP) published a set of widely used definitions for sarcopenia [5], which was updated in 2019 as EWGSOP2 [6]. The revised diagnostic criteria specify that sarcopenia is present when there is a decline in muscle mass and/or quality plus low muscle strength. The commonly used diagnostic cutoff values are the appendicular muscle mass index less than 7.0 kg/m^2^ for men or 5.5 kg/m^2^ for women, or hand-grip strength below 27 kg for men or 16 kg for women. On the other hand, the International Working Group on Sarcopenia (IWGS) defines sarcopenia based on low muscle mass, with thresholds of ≤ 7.23 kg/m^2^ for men and ≤ 5.67 kg/m^2^ for women, together with a low gait speed of ≤ 1 m/s [7]. The concordance between sarcopenia definitions by EWGSOP and IWGS is only moderate, resulting in considerable variation in prevalence rates between different studies [8].

### 2.2. Pathophysiology of Sarcopenia

The pathogenic factors for sarcopenia in the general elderly population, CKD, and PD patients are outlined in Table 1. In the general population, aging is the primary cause of sarcopenia. Simply put, aging disrupts the balance between muscle protein synthesis and degradation, leading to an overall loss of skeletal muscle [18].

Aging is associated with reduced dietary intake and physical activity, both of which may contribute to the development of sarcopenia. Specifically, aging diminishes the function of muscle satellite cells, resulting in a reduced regenerative capacity and a lower number of myofibers [29, 30]. Additionally, aging is linked to a decrease in motor units [31, 32].

Furthermore, the elderly exhibit significantly elevated inflammatory cytokine levels, notably tumor necrosis factor-alpha (TNF-α) and interleukin-6 (IL-6) [22]. In mice models, TNF-α and IL-6 drive skeletal muscle degradation [33, 34]. Inflammatory cytokines also lead to mitochondrial dysfunction, reduced ATP production, and increased reactive oxygen species (ROS) [24, 25]. The ubiquitin–proteasome system (UPS) is consequently activated, promoting proteolysis and muscle atrophy [26].

In addition to aging, patients with CKD have various physiological stresses that contribute to muscle loss. Chronic inflammation and oxidative stress are particularly pronounced in CKD [27, 28]. IL-6 and TNF-α are further elevated in CKD patients, leading to aggravated muscle loss [23]. In addition, heightened oxidative stress results in the oxidation of essential biomolecules, further contributing to muscle wasting [28]. In murine CKD models, myogenic factors secreted by satellite cells are reduced, which also leads to the suppression of myogenesis [35]. In human CKD, the synthesis rates of mixed muscle protein and myosin are 27% and 37% lower than those of healthy individuals [19]. Metabolic acidosis, which is common in CKD, increases the expression of ubiquitin mRNA and proteasome subunits, which leads to increased muscle protein degradation [20, 21].

MicroRNAs also play a significant role in muscle wasting associated with CKD, which is associated with decreased levels of miR-29a and miR-29b, both of which suppress the expression of YY1, a protein that inhibits myogenesis [36]. In mice with CKD, miR-23a, which protects muscles from atrophy, is also reduced [37].

Other mediators contribute to sarcopenia by promoting muscle breakdown or inhibiting myogenesis. Irisin and insulin-like growth factor 1 (IGF-1) are pro-myogenic [38, 39]; reduction in these factors correlates with decreased muscle growth [40, 41]. In contrast, myostatin inhibits muscle growth, and elevated myostatin levels are observed in patients with muscle loss [42]. Similarly, activin A contributes to sarcopenia as it shares cellular pathways with myostatin, while growth differentiation factor-15 (GDF-15) has catabolic effects on muscle cells, potentially through the downregulation of muscle-protective microRNAs [43]. Increased serum GDF-15 concentration is associated with increased muscle degradation and the incidence of sarcopenia [44]. Cortisol also contributes to sarcopenia by reducing glucose uptake and enhancing protein degradation in muscle cells [45].

For patients undergoing PD, malnutrition and anorexia are important causes of sarcopenia [11]. Glucose absorption from PD solution diminishes appetite and oral nutrient intake [12], leading to decreased myogenesis and muscle degeneration [46]. Many PD patients do not respond well to nutritional supplements because of gastrointestinal disease or underlying systemic inflammation [13]. Previous studies further showed that patients with a high peritoneal transport state had more protein loss into the dialysate, lower serum albumin, and increased muscle loss [14]. Under ordinary conditions, protein loss during PD ranges from 5 to 15 g per day [47], but it is much higher during peritonitis episodes [48].

## 3. Muscle Markers

In addition to measuring muscle mass and strength directly, a number of circulating markers have been explored to serve as surrogate indicators of sarcopenia. Broadly speaking, they could be classified into myokines, adipokines, hormones, and other muscle markers.

### 3.1. Myokines

Myokines are protein factors secreted by skeletal muscle cells and exert autocrine, paracrine, or endocrine effects. Several protein factors, such as myostatin, follistatin, activin A, GDF-15, and irisin, are specifically produced by skeletal muscles and are often considered as “conventional” myokines [49]. Other factors, such as IGF-1, IL-6, and TNF-α, are secreted by many cell types, and muscle cells contribute only a small fraction of the circulating level. In general, myokines play pivotal roles in the regulation of muscle metabolism, angiogenesis, and inflammatory response [50]. Measuring the circulating myokine levels may help evaluate the muscle mass and identify sarcopenia. In addition, myokines are also produced by cardiomyocytes, and dysregulation of myokines in CKD has been linked to an increased risk of heart failure due to adverse cardiac remodeling and poor outcomes [49], which is outside the scope of this review.

#### 3.1.1. Myostatin

Myostatin, a member of the transforming growth factor-β (TGF-β) superfamily, plays a crucial role in the regulation of muscle growth by binding to activin types IIA (ActRIIA) and IIB (ActRIIB) receptors, thereby inhibiting mammalian target of rapamycin (mTOR)–mediated protein synthesis [50]. In addition, myostatin suppresses the proliferation and differentiation of satellite muscle cells and can induce a proteolytic phenotype in muscle cells through the UPS [51].

Despite its well-documented role in muscle growth suppression, the evidence surrounding its functions is conflicting. In dialysis and predialysis CKD patients, serum myostatin levels correlated positively with muscle mass, skeletal muscle index, and hand-grip strength, supporting its role as a muscle mass marker [52–54]. One study further showed that myostatin level was associated with the 1-year mortality rate in HD patients [52]. However, other studies present contradictory findings. Elevated myostatin expression in skeletal muscle cells and increases in circulating myostatin levels have been reported in CKD [49, 51] and found to be linked to muscle atrophy [42]. An animal study showed that myostatin inactivation enhanced muscle mass and strength in mouse models of sarcopenia [55]. A study in dialysis and predialysis CKD patients also reported a negative association between myostatin levels and hand-grip strength [56]. The dichotomous findings highlight the necessity for further investigation into the mechanisms and effects of myostatin, particularly its correlation with muscle dynamics and sarcopenia.

#### 3.1.2. Follistatin

Follistatin, a myokine, functions as an antagonist to myostatin by influencing myogenic factors such as MyoD, Myf5, and myogenin [57, 58]. The relationship between follistatin and muscle mass, however, remains a subject of debate. In a cross-sectional study involving older adults, serum follistatin levels were positively correlated with muscle strength and muscle mass in women [59]. In contrast, a negative correlation between circulating follistatin levels and muscle strength was observed in patients with CKD [60]. Recent animal studies suggest that follistatin exhibits an inverse U-shaped expression pattern as sarcopenia progresses, which is in contrast to myostatin [61]. This nonlinear relationship may also be present in humans, potentially accounting for the inconsistencies observed in earlier studies. It is noteworthy that no studies have investigated the relationship between follistatin level and muscle mass in dialysis patients.

Recent studies highlighted the potential clinical implications of circulating follistatin. Elevated blood follistatin levels are associated with an increased risk of heart failure, stroke, and all-cause mortality in the Malmö Diet and Cancer Study Cardiovascular Cohort (MDC-CC) [62]. Similarly, in the general elderly population, elevated follistatin levels correlated with impaired pulmonary function, and the coexistence of both conditions was an independent risk factor for all-cause mortality [63]. On the other hand, in CKD patients, follistatin exhibits renal-protective properties by neutralizing ROS, thereby preventing apoptosis of glomerular mesangial cells and mitigating the progression of CKD [64]. Miyamoto et al. found that serum follistatin levels did not correlate with the mortality rates of CKD patients [60], while a prospective cohort study reported that HD patients with lower baseline follistatin levels had a significantly reduced risk of death [65].

Given that myostatin and follistatin have opposing functions, the ratio of myostatin to follistatin may offer greater clinical significance. Previous research has indicated that this ratio in peripheral blood could serve as a potential biomarker for denervated muscle atrophy, another form of muscle wasting [66]. However, their role in sarcopenia and among dialysis patients remains unexplored.

#### 3.1.3. Activin A

Activin A also belongs to the TGF-β family. It binds to the ActRIIB receptor [67] and negatively influences muscle growth [68]. The activin–myostatin–follistatin system plays a vital role in regulating muscle and bone mass [69, 70]. In chronic obstructive pulmonary disease (COPD) patients, higher serum activin A levels correlate with muscle wasting [71]. In critically ill patients, elevated activin A levels were associated with declining physical function [72]. However, in inflammatory bowel disease, sarcopenic patients had lower activin A levels than nonsarcopenic ones [73]. In mouse models of CKD, activin A levels were elevated and were related to progressive muscle wasting [74]. In human CKD, serum activin A level was significantly increased [75], but the relation with muscle mass or function has not been well studied.

The clinical implications of activin A, however, are controversial. Elevated activin A levels were identified as an independent risk factor for mortality in cancer patients, probably related to cachexia and skeletal muscle loss [76]. In CKD, serum activin A levels negatively correlated with kidney function [75] and may contribute to fibrosis and inflammation in CKD [77]. However, the prognostic role of serum activin A in CKD or dialysis populations has not been studied.

Pharmacological blockade of activin A is now available and has been shown to reduce muscle loss in mouse models of CKD [74, 78]. Bimagrumab, a monoclonal antibody against ActRIIB receptor, has promising preliminary results on muscle mass and exercise performance in sarcopenic patients [79, 80].

#### 3.1.4. GDF-15

GDF-15, part of the TGF-beta superfamily, is induced under stress conditions [81] and is secreted by various cell types, including skeletal muscle cells, cardiomyocytes, and vascular smooth muscle cells [82]. In the brain stem, GDF-15 binds to its receptors, triggering aversive effects such as anorexia and nausea [83].

Several studies highlight the link between serum GDF-15 levels and muscle wasting. High GDF-15 levels are associated with reduced muscle performance and increased inflammation [84]. Inverse correlations between GDF-15 level and skeletal muscle index or muscle strength were reported in patients with Crohn's disease [44], cardiometabolic diseases [85], and those awaiting cardiovascular surgery [86] or undergoing aortic valve replacement [87]. Combining GDF-15 with myostatin levels reliably predicts sarcopenia [88]. In hemodialysis patients, GDF-15 levels inversely correlated with serum creatinine, and higher GDF-15 levels were associated with major adverse cardiac events and increased mortality in some studies [89–91] but not observed by other groups [92, 93]. Taken together, the relationship between GDF-15 and sarcopenia or clinical outcomes remains controversial and has not been explored in PD patients. Nonetheless, the importance of GDF-15 in sarcopenia has a particular topical interest [94]. Ponsegromab, a humanized monoclonal antibody targeting GDF-15, has recently reported to promote weight gain and increase lumbar skeletal muscle index and physical activity in cancer patients with cachexia [95]. However, its effects on muscle mass in CKD or dialysis patients need further study.

#### 3.1.5. Irisin

Irisin is a myokine that is secreted following proteolytic cleavage of its precursor fibronectin type III domain containing 5 (FNDC5), which is a Type I transmembrane glycoprotein. Irisin plays a crucial role in the conversion of white adipose tissue to beige adipose tissue [96].

Several studies reported a positive association between irisin level and muscle mass [38, 97]. A recent meta-analysis indicated that circulating irisin levels are lower in sarcopenic patients, correlating with muscle mass and strength, but not muscle function (e.g., gait speed or chair test time) [98]. Further studies demonstrated that serum irisin level, in conjunction with phase angle or other clinical indicators, predicted the risk of sarcopenia in PD patients [40, 99].

Serum irisin levels are also associated with various adverse health events. In dialysis patients, irisin contributes to the maintenance of insulin sensitivity, inhibition of bone resorption, and prevention of vascular calcification [100]. Patients with CKD typically have lower skeletal muscle expression of irisin and circulating irisin levels than healthy individuals [49, 101]. Lower serum irisin levels are associated with increased abdominal aortic calcification in PD patients [102, 103]. Observational studies further reported that baseline irisin levels independently predicted cardiovascular events in CKD patients [102, 103] and cardiovascular mortality, but not all-cause mortality, in PD patients [104]. Irisin supplementation has been shown to enhance protein synthesis and myogenesis in mice [38]. Further human studies are needed to confirm the findings.

#### 3.1.6. IGF-1

IGF-1 is secreted by many tissue types, including skeletal muscle, and could be considered a myokine. However, the specificity of IGF-1 to muscle is low [50]. It has anabolic properties, and the expression is induced by physical stress [105]. Notably, IGF-1 facilitates protein synthesis in skeletal muscle via the PI3K/Akt/mTOR and PI3K/Akt/GSK3β pathways [106]. In mice models of CKD, impaired IGF-1 signaling has been shown to cause satellite cell dysfunction and muscle atrophy [35]. Several studies have shown that serum IGF-1 levels correlated with muscle mass and function in humans, particularly in middle-aged and elderly adults [41, 93, 107]. In CKD patients, skeletal muscle expression of IGF-1 is decreased [49], but the circulating IGF-1 levels were not consistently altered [108, 109]. Low levels of IGF-1 in CKD are linked to reduced muscle strength and an increased risk of all-cause mortality, especially in patients with diminished hand-grip strength [110]. Kwak et al. reported that serum IGF-1 levels are significantly lower in sarcopenic patients [41]. Utilizing IGF-1 in conjunction with other serum markers, they developed a predictive model with high sensitivity and specificity for the diagnosis of sarcopenia [41]. However, the clinical significance of IGF-1 and its relation with sarcopenia in dialysis patients remain unexplored.

### 3.2. Other Muscle Markers

In addition to myokines, other circulating factors have been reported to serve as biomarkers of muscle mass or function. Although adipokines are produced by adipocytes, the cross-talk between muscle cells and adipocytes is well-established [111]. Several adipokines are involved in the maintenance of muscle mass [112], and the circulating level of several adipokines may serve as markers of sarcopenia [113].

#### 3.2.1. Adiponectin

Adiponectin is crucial in insulin resistance and inflammation regulation. Though primarily produced by adipocytes, it is also produced in skeletal muscle [114]. In mice, adiponectin promotes muscle regeneration [115], and AdipoRon, an adiponectin receptor agonist, enhances muscle function in male mice [116].

Conflicting findings exist regarding adiponectin levels and human sarcopenia. Harada et al. reported significantly higher serum adiponectin levels in CVD patients with sarcopenia, as compared to nonsarcopenic ones [117]. Several studies reported a negative correlation between adiponectin level and muscle mass, quality, or function in obese patients [118–120]. Conversely, Li et al. suggested a lower circulating adiponectin level was linked to increased risk of sarcopenia [121]. A meta-analysis concluded that sarcopenic patients had elevated adiponectin levels [122]. In CKD patients, Verzola et al. noted adiponectin overexpression in muscle, while its receptor levels were significantly reduced, which may explain the promyogenic potential of adiponectin despite inverse associations with muscle mass [123]. In dialysis patients, a recent predictive model that included age, albumin, adiponectin, and myostatin levels could reliably identify the presence of sarcopenia [124].

#### 3.2.2. Leptin

Unlike adiponectin, leptin functions as a proinflammatory adipokine and exhibits antagonistic effects relative to adiponectin [112]. Owing to its association with inflammation [125], elevated leptin levels may contribute to muscle wasting by inhibiting protein synthesis in muscle cells. Nonetheless, there are conflicting findings regarding the relationship between leptin levels and muscle mass or strength. Hamrick et al. [126] reported that the administration of recombinant leptin significantly increased skeletal muscle mass and upregulated the expression of myogenic miRNA in aged mice. Li et al. reported that serum leptin levels were significantly higher in sarcopenic patients than in the healthy population [121], while another study found that elderly patients with sarcopenia had significantly reduced serum leptin levels after adjusting for adipose tissue mass [127]. Serum leptin-to-adiponectin ratio, given the counteracting effect of the two adipokines, significantly correlated with hand-grip strength in the healthy population [128]. In cancer patients with cachexia, changes in serum leptin levels correlated with muscle mass and strength [129].

In hemodialysis patients, low serum leptin levels were independently associated with sarcopenia [130], and serum leptin levels inversely correlated with lean tissue mass [131]. In contrast, Hsu et al. showed that serum leptin levels had a positive correlation with skeletal muscle index but an inverse correlation with hand-grip strength and muscle quality in male hemodialysis patients [132]. The role of leptin level or leptin-to-adiponectin ratio as a muscle marker in PD patients, however, remains unexplored.

#### 3.2.3. Vitamin D

Vitamin D is an essential nutrient and plays a crucial role in bone and skeletal muscle health. The biologically active form, 1,25-dihydroxycholecalciferal (1,25-OH_2_D_3_), is synthesized in the kidneys. Recent studies indicate that vitamin D is instrumental in promoting skeletal muscle regeneration by binding to vitamin D receptors (VDRs) on muscle fibers [133]. The downstream effects on skeletal muscle include enhancement of progenitor skeletal muscle cell migration, improvement of muscle fiber cross-sectional area, reduction of myostatin expression, and elevation of follistatin levels [134]. Low serum vitamin D levels are associated with loss of muscle mass and strength [135]. In addition to the fully active 1,25-OH_2_D_3_, a recent study reported that 25-hydroxycholecalciferal (25(OH)D) was an independent risk factor for sarcopenia in HD patients [136].

The effects of vitamin D supplements on muscle health, however, remain a topic of debate. One study reported that a daily supplement of calcifediol 20 μg enhanced muscle strength and physical performance in postmenopausal women [137], while another randomized controlled trial showed that 25(OH)D supplementation did not affect muscle mass or strength in individuals with vitamin D deficiency [138]. In the context of CKD, a comparative study showed that vitamin D supplementation alone was not sufficient to improve muscle strength, but significant improvement was observed when the supplement was combined with physical training [139]. In dialysis patients, Singer et al. reported that cholecalciferol supplementation did not affect muscle strength [140]. However, another study found that treatment with calcitriol or paricalcitol was associated with increased muscle mass and strength, but not with physical function, in HD patients [141], and vitamin D replacement therapy significantly improved muscle strength and function in PD patients with vitamin D deficiency [142]. In another retrospective study on PD patients without overt vitamin D deficiency, users of calcitriol or paricalcitol had a significantly lower risk of deterioration in muscle mass and function than nonusers [143]. Taken together, there are uncertainties regarding the relation between vitamin D status and sarcopenia, as well as the role of vitamin D supplements on the muscle health of dialysis patients.

#### 3.2.4. Myoglobin

Myoglobin is a muscle protein with a specific affinity for binding iron and oxygen [144]. In healthy individuals, serum myoglobin levels are typically very low. However, these levels may significantly increase following muscle injury [145]. A previous study showed that plasma myoglobin levels are elevated in CKD patients, and a high plasma myoglobin level has been reported to be associated with increased overall and cardiovascular mortality [146]. Serum myoglobin level has been proposed to be a marker for muscle breakdown, but published data in this area are scarce.

#### 3.2.5. Cortisol

Cortisol plays critical roles in the regulation of many metabolic processes [147]. Notably, due to its catabolic properties, cortisol is linked to muscle loss [148]. Yanagita et al. demonstrated that an elevated cortisol-to-dehydroepiandrosterone sulfate (DHEA-S) ratio is an independent risk factor for sarcopenia in elderly diabetic patients [149]. Another study reported that increased salivary cortisol levels are associated with sarcopenia in postmenopausal women [150]. Nonetheless, there is a circadian rhythm of serum cortisol level, which makes it challenging to be used as a marker of muscle mass.

## 4. Conclusion and Directions for Future Studies

**Figure 1:**
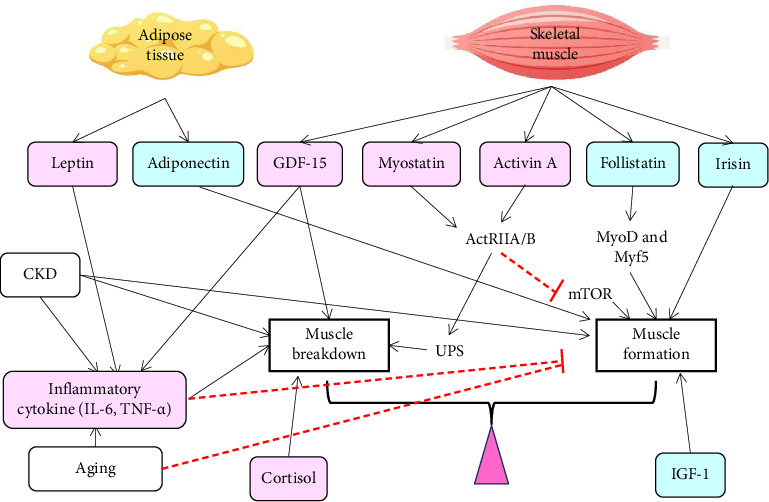
Schematic overview on the effects of myokines, adipokines, and other hormonal factors on muscle homeostasis. Pink boxes indicate mediators that promote muscle breakdown, while blue boxes indicate mediators that favor muscle formation. Black arrows indicate positive stimulation, red arrows with blunt ends indicate inhibition. Insulin-like growth factor-1 (IGF-1), interleukin-6 (IL-6), and tumor necrosis factor-alpha (TNF-α) are secreted by muscle cells and many other cell types, and muscle cells contribute only to a small fraction of the circulating level. CKD, chronic kidney disease; GDF-15, growth differentiation factor 15; UPS, ubiquitin–proteasome system; ActRIIA/B, activin receptors type IIA and IIB; mTOR, mammalian target of rapamycin.

This article reviews the current literature on biomarkers of muscle mass and function, emphasizing their clinical relevance in patients undergoing PD. Sarcopenia is a complex condition influenced by aging, kidney disease, and PD, with muscle homeostasis governed by a dynamic interplay of myokines, adipokines, and hormonal factors (Figure 1). Key mediators such as IGF-1, myostatin, activin A, and inflammatory cytokines play pivotal roles in either promoting muscle growth or driving muscle breakdown. CKD and aging accelerate muscle loss through mechanisms, including malnutrition, physical inactivity, inflammation, oxidative stress, and disrupted protein metabolism. These processes are particularly exacerbated in PD patients due to additional nutrient losses and exposure to bioincompatible dialysate. Although our understanding of these molecular pathways continues to advance, significant gaps remain in applying this knowledge effectively in clinical practice.

Future research should focus on identifying and validating muscle biomarkers that accurately reflect the progression of sarcopenia and responses to therapy (see Table 2). Laboratory studies are necessary to clarify the pathogenic roles of these biomarkers, while standardizing assay methods will improve reproducibility across clinical settings. The development of point-of-care diagnostic tools, along with integration with other assessment methods, may enable earlier detection and more personalized treatments. Large-scale validation studies involving diverse populations, including direct comparisons between hemodialysis and PD patients, are essential. Furthermore, prospective and interventional trials are needed to establish causal relationships and assess therapeutic potential. Ultimately, advancing sarcopenia research and improving patient outcomes will require a multidisciplinary approach that combines molecular biology, clinical nephrology, and geriatric medicine.

## Figures and Tables

**Table 1:** Pathogenic factors for sarcopenia across general elderly, CKD, and PD patients.

Pathogenic factors	General elderly	CKD	PD
Nutritional deficiency	Reduced dietary intake of proteins due to reduced appetite caused by aging [9]	Anorexia caused by CKD may further reduce the intake of essential nutrients; also a component that reduces protein synthesis [10].	Glucose absorption from dialysate further reduces appetite [11–13]; nutrient loss to the dialysate [14].
Physical inactivity	Aging causes reduced physical activity; indirectly causing decreased appetite and protein synthesis [15, 16]	Fatigue and anemia in CKD patients further decreased physical activity and reduced myogenesis [17].	Similar to CKD patients but generally more severely affected.
Imbalanced muscle protein metabolism	More protein degradation than synthesis due to aging [18]	Reduced kidney function aggravates muscle breakdown and reduces myogenesis [19]. Metabolic acidosis is highly common in CKD, which further accelerates muscle loss [20, 21]	Similar to CKD patients but generally more severely affected.
Inflammation	Increased basal levels of inflammatory cytokines (IL-6, TNF-α) with aging, which increase muscle breakdown [22]	Persistent low-grade inflammation is common in CKD [23]	Persistent low-grade inflammation [23] often complicated with intercurrent infections and low-grade inflammation in response to bioincompatible PD solutions.
Oxidative stress	Age-related mitochondrial dysfunction and impairment in antioxidant defense result in elevated ROS levels, which contributes to muscle loss by oxidation of essential biomolecules [24–26]	ROS levels are typically increased in CKD patients [27, 28]	Similar to CKD patients.

Abbreviations: CKD, chronic kidney disease; IL-6, interleukin-6; PD, peritoneal dialysis; ROS, reactive oxygen species; TNF-α, tumor necrosis factor-alpha.

**Table 2:** Potential areas of further research on muscle biomarkers.

⁃ Laboratory research to understand their role in the pathogenesis of sarcopenia
⁃ Standardization of their assay technique
⁃ Development of point-of-care tests
⁃ Validation studies in larger and diverse populations
⁃ Integration of muscle markers and other diagnostic tools
⁃ Prospective studies
⁃ Interventional trials (or Mendelian trials)
⁃ Comparison of their utility between hemodialysis and peritoneal dialysis
